# Rescue of bacterial motility using two- and three-species FliC chimeras

**DOI:** 10.1128/jb.00517-24

**Published:** 2025-08-11

**Authors:** Jacob Scadden, Pietro Ridone, Divyangi Pandit, Yoshiyuki Sowa, Matthew A. B. Baker

**Affiliations:** 1 School of Biotechnology and Biomolecular Sciences, University of New South Wales 7800, Sydney, Australia; 2 Department of Frontier Bioscience, Hosei University 12814, Tokyo, Japan; 3 Research Center for Micro-Nano Technology, Hosei University 12814, Tokyo, Japan; National Institutes of Health, Bethesda, Maryland, USA

**Keywords:** flagella, *Escherichia coli*, flagellar structure, flagellar motility, protein structure-function, motility

## Abstract

The bacterial flagellar filament acts as a propeller to drive most bacterial swimming. The filament is made of flagellin, known as FliC in *Escherichia coli*. FliC consists of four domains, the highly conserved core D0 and D1 domains and the hypervariable outer D2 and D3 domains. The size and structure of the outer domains vary, being completely absent in some bacterial species. Here, we sought to identify outer domains from various species that are compatible with the ability of *E. coli* K-12 FliC to form filaments capable of supporting motility. We calculated a phylogeny of 210 representative flagellin amino acid sequences and generated a series of FliC variants, including outer domain-deleted forms and 11 chimeric FliC mutants using domains from *E. coli K-12*, *Salmonella* Typhimurium, *Pseudomonas aeruginosa*, *Collimonas fungivorans*, *Helicobacter mustelae*, and *Mesorhizobium* sp. ORS3359 in various combinations. Notably, two of the chimeric *fliC* mutants rescued motility in a *fliC*-disrupted *E. coli* K-12 strain, both of which contained the *S*. Typhimurium D2 domain. Overall, we demonstrate that, while most FliC chimeras did not support motility, interchangeability of the outer domains can produce filaments that provide motility, providing insights to guide the design of synthetic flagellins.

**IMPORTANCE:**

Flagellin is a key protein forming the filament of the bacterial flagellar motor which powers most bacterial swimming. Flagellin can have hypervariable domains which can alter motility in different environments and provide immune evasion. Here we engineered two flagellin chimeras that could drive motility. This indicates that the flagellin outer domains can be exchanged, to some degree, allowing us to refine rational design approaches for engineering of bacterial swimming. Our work shows the challenges to overcome when combining flagellins from different species and provides evidence that domain-switched flagellins can form filaments.

## INTRODUCTION

Motility provides organisms the ability to exploit new niches, to evade predation, and to find increased opportunities for the exchange of genetic material (1). The flagellar filament is a major component of the bacterial flagellar motor, taking the form of the bacterial flagellum that consists of a long helical protein filament, which is formed from approximately 30,000 flagellin subunits, known as FliC in *Escherichia coli* K-12 (2, 3). FliC interacts with both the flagellar hook junction proteins (FlgK and FlgL) and filament cap protein (FliD) (4–6).

FliC from *E. coli* K-12 is composed of four domains: D0, D1, D2, and D3 (7, 8). Domains D0 and D1 are found on the N- and C-termini of the protein and are highly conserved across multiple bacterial phyla, such as *Proteobacteria*, *Bacillota*, and *Actinobacteria* (9). These “core” domains are formed of α-helices and interact with each other as part of the supercoiled filament (7, 8, 10, 11). Within the D0 domain is a 22-residue segment, which is the recognition signal for the flagellar export chaperone, FliS (12). Domains D2 and D3 in *E. coli* K-12, also termed “outer domains,” are variable in both sequence and length even at a species level (3). These outer domains consist primarily of β-sheets and have been shown in *Salmonella* Typhimurium to have no interactions between each other (7, 13). In some cases, flagellin monomers from other bacterial species such as *Bacillus cereus*, *Agrobacterium tumefaciens*, and *Treponema pallidum* do not contain a D2 or D3 domain but still produce a functional flagellar filament capable of motility (9, 14–18). It has been shown that within the genera *Escherichia* and *Salmonella*, there is a large level of diversity in the outer domains, suggesting that the outer domains are acquired via the process of evolution (19, 20). This implies that the outer domains of flagellin from diverse species can be incorporated into host flagellins to provide competitive advantages that increase efficiency of motility in different environments or that allow evasion of recognition by the immune system.

In *Pseudomonas aeruginosa* PAO1, the outer domains of flagellin increase the motility of bacteria in more viscous environments due to inter-domain interactions (21). Likewise, the outer domains *E. coli* O157:H7, *E. coli* O127:H6, *Achromobacter* (which have an additional “D4” domain), and *Sinorhizobium meliloti* (which has only a D2 outer domain) can dimerize or tetramerize to form a screw-like surface which increases motility through stabilization of an intermediate waveform and prolonging tumbling (22). Furthermore, interactions of the outer domains of *E. coli* can increase tumbling time (22). Inter-species flagellin chimeras between *Salmonella* strains (serovar Typhimurium strain 14028s and serovar Enteritidis strain P125109) were able to form functional filaments (23). Of 13 FliC variants, 4 were motile, and it was shown that alternating the outer domains could impact motility (23).

One of the main research focuses on the flagellin outer domains is their use in immune activation and protein display. *S*. Typhimurium FliC is capable of activating Toll-Like Receptor 5 (24). In a comprehensive review by Hajam et al., the authors identified 29 studies where flagellin was used as an immune adjuvant for bacterial, viral, parasitic, and other miscellaneous antigens (25). This use of recombinant flagellin as a surface-display tool for immune activation has led to a number of vaccines being developed and used in human clinical trials (26, 27). However, although chimeric FliC has been used in immunological studies, the effect of manipulating the outer domains on bacterial motility is not well understood.

Here, we tested the ability of chimeric FliC to support motility, where we replaced the outer domains of *E. coli* K-12 with outer domains from flagellins of a diverse set of bacteria. We show that the outer domains of *E. coli* K-12 FliC are not required to drive swimming motility and that two forms of chimeric FliC can form functional filaments that support swimming motility.

## MATERIALS AND METHODS

### Bacterial strains, plasmids, and growth conditions

Table S1 describes the strains and plasmids used in this study. The *E. coli* strain SYC29 was constructed from the chemotactic wild-type strain RP437, using the λ RED recombination system, as previously described (28, 29). *E. coli* strains were grown in lysogeny broth (LB) (10 g/L NaCl, 10 g/L Bacto tryptone, and 5 g/L yeast extract). For 2% (wt/vol), 0.3%, 0.35%, and 0.4% (wt/vol) LB agar plates, 20.0, 3.0, 3.5, or 4.0 g/L agar was added, respectively. *E. coli* strains were grown in tryptone broth (TB) to aid in induction of motility (5 g/L NaCl, 10 g/L Bacto tryptone). Antibiotic selection used ampicillin (AMP) (50 µg/mL), chloramphenicol (CAM) (25 µg/mL), and/or tetracycline (TET) (10 µg/mL). Induction for gene expression from plasmids required L-arabinose (0.02% [wt/vol]) and/or isopropyl β-D-1-thiogalactopyranoside (0.1 mM).

### Phylogenetic analysis of FliC

To investigate the structural diversity of the outer domains of flagellins and their presence/absence across the tree of life, eight flagellin amino acid sequences (Table S2) were used as reference sequences for a BLASTP search in UniProt (30) (*E*-threshold, 10; matrix, BLOSUM62; filter, none; gapped, yes; hits, 1,000; High-Scoring Segment Pairs per hit, all). All hits for each BLASTP search were selected and used in ID mapping from database UniProtKB AC/ID to UniRef90 (31). The clusters were sorted by member size, and each list was manually curated to select 210 representative flagellin sequences for further phylogenetic analysis, in addition to 14 FliD amino acid sequences as an outgroup. The 224 sequences were downloaded in FASTA format and uploaded to CIPRES Science Gateway (32). Alignment of the representative sequences was performed using MUSCLE (v.3.7) (33) and viewed using AliView (34). The alignment was used to generate a phylogenetic tree using IQTree (v.2.3.2) (35) using CIPRES (for parameters, see Table S3) (32). A total of 682 representative flagellins were downloaded from the supplementary information from Fields et al. (20); 605 were available on the Uniprot database and the amino acid sequences used in a MUSCLE alignment (33). A FastTree2 (36) phylogenetic tree was generated using CIPRES (32). Phylogenetic trees were visualized and annotated in ItoL (v.6) (37).

### Design and structural prediction of chimeric FliC

To study the effect of outer domain manipulation on bacterial motility, a rational design approach for each chimeric FliC candidate was taken. It was decided to maintain the D0 and D1 domains of *E. coli* K-12 FliC (UniProt P04949) (EEEE) in order to preserve the export recognition sequence found in the D0 domain (12). Therefore, to understand if these outer domains are functionally required for flagellum formation, the first *E. coli* K-12 *fliC* variants designed were the *fliC_*Δ*D3* (EEE) and *fliC_*Δ*D2/D3 (EE*), with deletions of residues 195–300 and 174–405, respectively. For the two-species and three-species FliC chimeras, five flagellin amino acid sequences were used from across the bacterial tree of life, *Helicobacter mustelae* (P50612), *Mesorhizobium* sp. ORS3359 (A0A090GIX0), *Pseudomonas aeruginosa* (P21184), *Collimonas fungivorans* strain Ter331 (G0AIL6), and *Salmonella* Typhimurium strain LT2 (P06179). The D2 and/or D3 domains of these flagellins were determined manually and used to replace the D2 and D3 domains of *E. coli* FliC. In the case of *S*. Typhimurium, additional chimeras were made using the D2 domain of *S*. Typhimurium and the D2 domains (in place of the D3 domains) of *Mesorhizobium* sp. ORS3359, *P. aeruginosa*, and *C. fungivorans* strain Ter331 were used. Structural prediction for all domain-deleted and chimeric FliC sequences was performed using AlphaFold3 (v.3.0.1) (38).

### Cloning of FliC chimeras into expression vector

Reverse translation of each FliC chimera from amino acid to nucleotide codon-optimized sequence for *E. coli* was performed in Benchling (Benchling Inc). Gene fragments for all chimeras and outer domain-deleted FliC were ordered using TWIST (Decode Bioscience) with EcoRI and NotI restriction sites incorporated in the 5′ and 3′ regions, respectively. Digestion of gene fragments and pET21(+) (Merck) expression vector was performed using EcoRI-HF and NotI-HF (NEB), followed by Antarctic phosphatase (NEB) treatment of pET21(+), as per the manufacturer’s instructions. Ligation was carried out using T4 ligase (NEB) as per the manufacturer’s instructions and incubated at room temperature overnight. After heat inactivation, the ligation mix was transformed into chemically competent *E. coli* 10-β (NEB) as per the manufacturer’s instructions. Transformations were incubated at 37°C for 1 h and plated on LB plates with AMP. Colonies were screened using pET21_F and pET21_R primers and Q5 Polymerase (NEB) (see Table S5 for primer details and PCR conditions). Positive colonies were grown overnight in LB medium with AMP, and plasmids were purified using a Qiagen Miniprep Kit, following the manufacturer’s protocol. Purified plasmid was sent for Sanger sequencing performed by the Ramaciotti Center for Genomics (UNSW, Sydney). The confirmed plasmids were then transformed into chemically competent *E. coli* SYC29 pDB108 (Δ*motAB*, *fliC::tetR*) and screened on LB plates containing AMP, CAM, and TET. This strain was selected due to its inducible motility characteristics. Positive colonies were screened using the same PCR method as previously described and grown overnight in LB medium containing AMP, CAM, and TET.

### Assessing the motility of chimeric FliC-expressing *E. coli*

Swim plate (0.30%, 0.35%, and 0.40% agar) motility assays were performed with required antibiotics and inducers as previously described (39). Plates were inoculated with 1 µL induced overnight culture and incubated at 30°C for 16 h. Plates were imaged using a Bio-Rad Gel Doc XR with Gel documentation system with analysis of Image Lab 6.0.1 (no filter, White Epi Illumination). Swim ring diameters were measured using ImageJ (FIJI) (40). Data were analyzed and displayed using GraphPad 2.0 (v.9.5.1).

### Free swimming assay for motile chimeric FliC-expressing *E. coli*

Mutant FliC-expressing *E. coli* spp. were grown overnight in LB medium containing appropriate antibiotics and inducers at 30°C. A 1% inoculum was grown in TB medium until OD_600_ 0.5. A tunnel slide was formed using tape and a glass coverslip, and 30 µL of sample was flown through, as previously described (41). Cells were visualized using a phase contrast microscope (Leica) and imaged using a Nikon camera. For each sample, 10 videos of 400 frames (20 frames/s) were recorded. Recordings were analyzed in a custom program in LabVIEW (National Instruments) (39) to quantify the swim speeds of each motile construct. GraphPad 2.0 (v.9.5.1) was used to analyze and display data.

### Flagella staining of chimeric FliC-expressing *E. coli*

Staining of flagellar filaments was performed using Remel Flagella stain (Thermo Fisher) as per the manufacturer’s protocol with minor changes. Strains of interest were inoculated on 0.3% LB agar plates, with appropriate antimicrobials and inducers, and incubated for 16 h at 30°C. To the edge of each swim ring, 30 µL of LB medium was added and incubated for 2 min at room temperature, after which 10 µL was spotted on a glass slide. After this step, the protocol was followed exactly. Samples were visualized using a Leica DM750 microscope at ×100 magnification, and images were captured using a MEE-500 camera (Yegren, China) and the camera app on Windows 10 (v.10.0.1.19045). Filament lengths were measured using ImageJ (FIJI) (40) using the freehand line tool. Data were analyzed and displayed using GraphPad 2.0 (v.9.5.1).

## RESULTS

### Flagellins cluster according to primarily phyla

**Fig 1:**
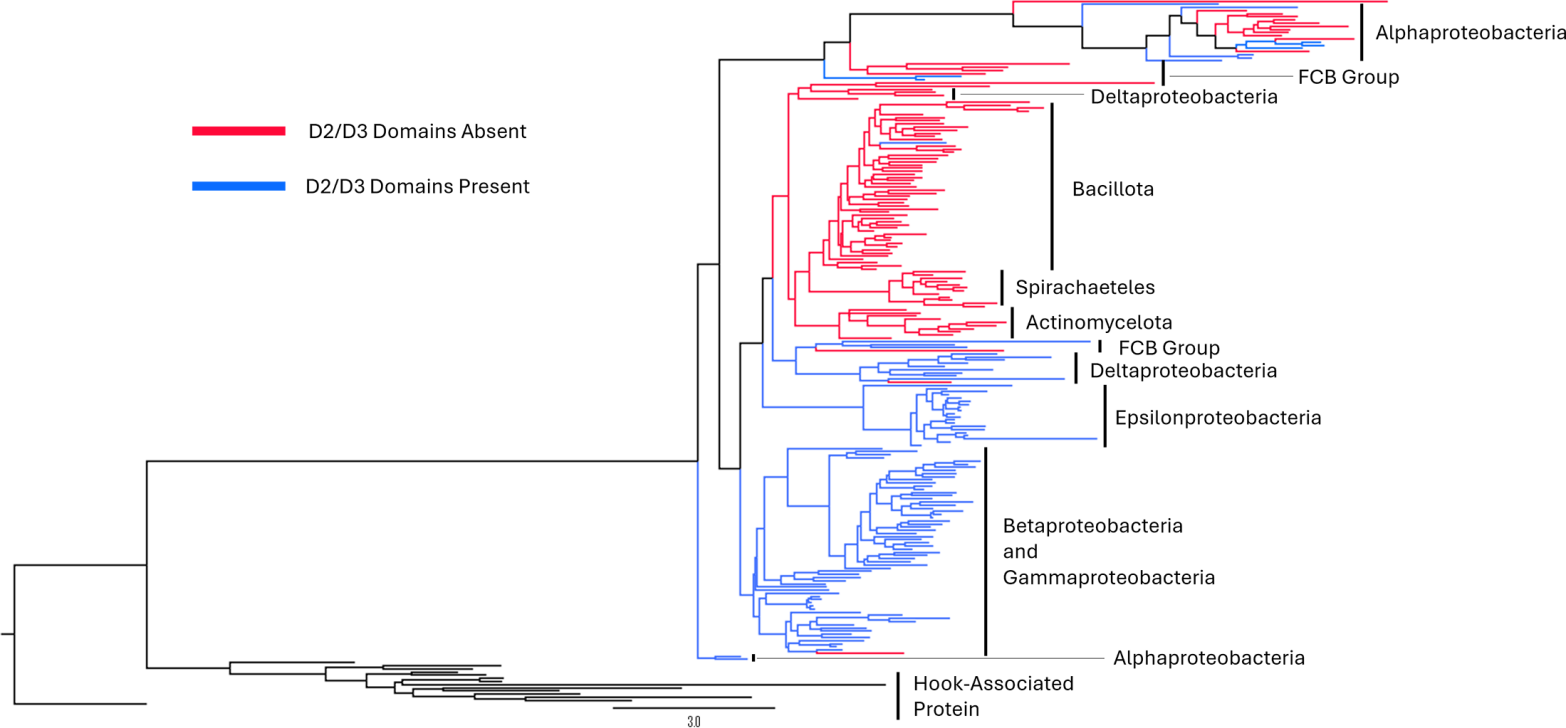
Phylogeny of 210 representative flagellin sequences, with 14 flagellar hook-associated proteins as an outgroup. Branches are colored based on the presence (blue) or absence (red) of the D2 and D3 domains.

To study the structural diversity of flagellin outer domains, 210 representative flagellin amino acid sequences from across the bacterial tree of life were selected, aligned, and used in the generation of a phylogenetic tree (Fig. 1). Of the representative flagellin sequences selected, 53% (112 out of 210) contained the outer D2 and D3 domains. The majority of the flagellins clustered according to the phyla or class to which the bacteria encoding the respective flagellin gene belonged, also seen in the phylogenetic analysis of 605 flagellin sequences from Fields et al. (20) (Fig. S1). In *Spirochaetota* (12 out of 12) and *Actinomycelotota* (10 out of 10), all flagellins did not contain the outer domains (Fig. 1, red branches). In *Bacillota*, none of the flagellins contained outer domains, with one exception (53 out of 54). The classes *Epsilonproteobacteria* (20/20) and *Betaproteobacteria*/*Gammaproteobacteria* (66/67) all clustered within their respective class and all contained the outer domains, with one exception (Fig. 1, blue branches). *Deltaproteobacteria* and the *Fibrobacteres-Chlorobi-Bacteroidetes* (FCB) superphylum had flagellin sequences both with and without outer domains, and the clading of these was split on the presence or absence of the outer domains. *Alphaproteobacteria* flagellins, in contrast, were the only group where sequences with and without the outer domains were mixed in the one clade (10 with and 12 without, respectively). In *Deltaproteobacteria* and the FCB superphylum, 64% (9 out of 14) and 42% (5 out of 12), respectively, contained outer domains.

The majority of flagellins containing outer domains were isolated from animal- and water-associated environments (Fig. S2A), whereas flagellins lacking the outer domains were found across soil, animal-associated, and aquatic environments (Fig. S2B). This was also observed when comparing isolation sources, with a higher percentage of flagellins that contained outer domains being isolated from animal and plant sources (63% and 55%, respectively) (Fig. S2C). However, flagellins lacking the outer domains were found predominantly in soil isolates (70%). There was a nearly equal percentage of flagellins containing outer domains and flagellins with no outer domains isolated from aquatic environments (51% and 49%, respectively). Within each phylum-based cluster, there was a minimal association of a flagellin type with a particular environment, with the exception of *Epsilonproteobactera*, 88% of which were isolated from animal sources, and *Alphaproteobacteria*, which were all either from aquatic or plant-associated environments.

### Outer domain-deleted and chimeric flagellin can rescue motility in *E. coli* K-12

**Fig 2:**
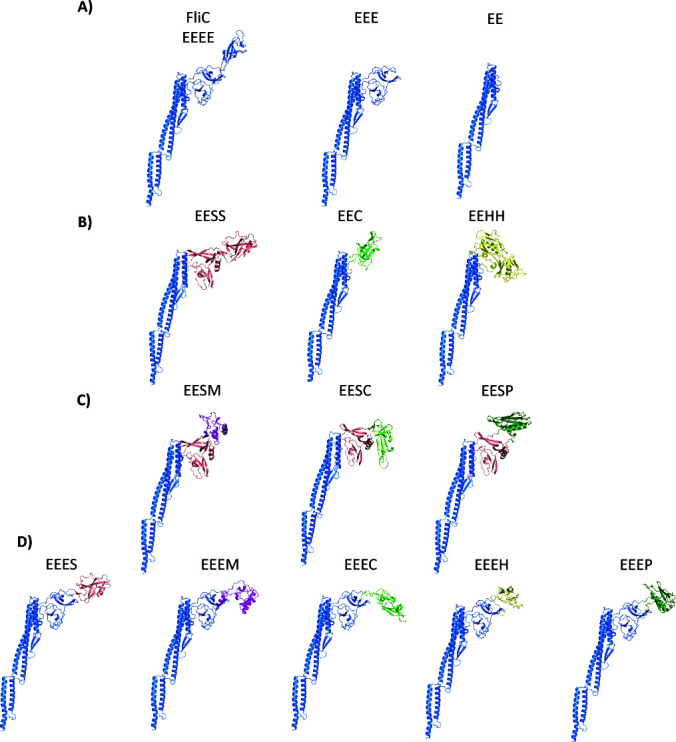
AlphaFold3 predictions of chimeric and outer domain-deleted *E. coli* K-12 FliC monomers designed for this study. (**A**) Predicted structures of wild type, D3-deleted, and D2/D3-deleted flagellin. (**B**) Predicted structure of *E. coli* K-12 D0 and D1 domains and single-species outer domains. (**C**) Predicted structures of *E. coli* K-12 D0 and D1 domains and multi-species outer domains. (**D**) Predicted structures of *E. coli* K-12 D0, D1, and D2 domains, with the D3 domain replaced with the outmost domain of a single-species flagellin. *E. coli* K-12 (blue), *H. mustelae* (yellow), *C. fungivorans* (light green), *S*. Typhimurium (pink), *Mesorhizobium* sp. OR3359 (purple), and *P. aeruginosa* (dark green).

First, to investigate the potential redundancy of the outer domains for bacterial motility, we deleted the nucleotide sequence coding for the outer domains from *E. coli* K-12 *fliC*. The nomenclature used for all flagellin outer domain deletions and chimeric variants created in this work represents each domain (D0–D3) as a single character of the first letter of the genus to which the flagellin is derived; thus, wild-type *E. coli* K-12 flagellin is termed EEEE. Structural prediction of these outer domain deletions indicated that sequential removal of these domains, removing D3 to generate EEE and both D2 and D3 to generate EE, would not affect the structure of the core domains (Fig. 2A, Fig. S3 and Table S4). All predicted aligned error scores indicated high confidence at the N- and C-termini with reduced confidence in the central section of the protein, correlating to the position of the outer domains (Fig. S4A through N). We then designed chimeric *fliC* variants using the D0 and D1 domains of *E. coli* K-12 and the outer domains of *S*. Typhimurium (EESS/EEES), *H. mustelae* (EEH/EEEH), and *C. fungivorans* (EEC/EEEC) (Fig. S3 and Table S4). The D0 and D1 domains were maintained as they are in *E. coli* K-12 to retain the export recognition site found between residues 26 and 47, given our use of *E. coli* K-12 proteins for all other elements of the motor, including flagellin export (12). *S*. Typhimurium (*Gammaproteobacteria*) (EESS/EEES) and *C. fungivorans* (*Betaproteobacteria*) (EEC/EEEC) were selected as members of the *Pseudomonadota* phylum, which clustered in the same clade as *E. coli* K-12 FliC (Fig. 1). The outer domains of *H. mustelae* (EEHH/EEEH), belonging to *Epsilonproteobacteria*, were selected due to being in a cluster where all members contained the outer domains (Fig. 1). The structures of these chimeric FliC variants were predicted using AlphaFold3, and these structural predictions showed that the tertiary structure of the core domains was conserved (Fig. 2B and D; Fig. S3 and S4).

In addition to direct replacement of the outer domain replacement with a domain from a single species, we decided to engineer three-species FliC chimeras. This was to assess the inter-compatibility of the outermost D3 domain. The D2 domain of *S*. Typhimurium was used for all D2 domains in these three-species FliC constructs (Fig. S3 and Table S4). The D3 domain was replaced with the outer domain from *C. fungivorans* strain Ter331 (EESC), *P. aeruginosa* (EESP), and *Mesorhizobium* sp. ORS 3359 (EESM) (Fig. 2C). The D3 domain from *C. fungivorans* strain Ter331 was chosen due to its FliC phylogenetic clading with the FliC from *E. coli*. Alternately, the D3 domain from *P. aeruginosa* and *Mesorhizobium* sp. ORS 3359 was chosen as previous work showed that the outer domains of *P. aeruginosa* PA01 and *Sinorhizobium meliloti* formed ridged filaments formed by interaction between the flagellin outer domains (Fig. 2C) (21, 22). Overall, chimeric and outer domain-deleted FliC ranged in size from 266 to 535 residues in length, with the outer D2 and D3 domains ranging from 107 to 269 amino acids in length (Table S4). Additionally, the outermost D3 domain of the *E. coli* K-12 flagellin was replaced with the outermost domain from *S*. Typhimurium, *Mesorhizobium* sp. ORS 3359, *C. fungivorans*, *P. aeruginosa*, and *H. mustelae* (Fig. 2D).

**Fig 3:**
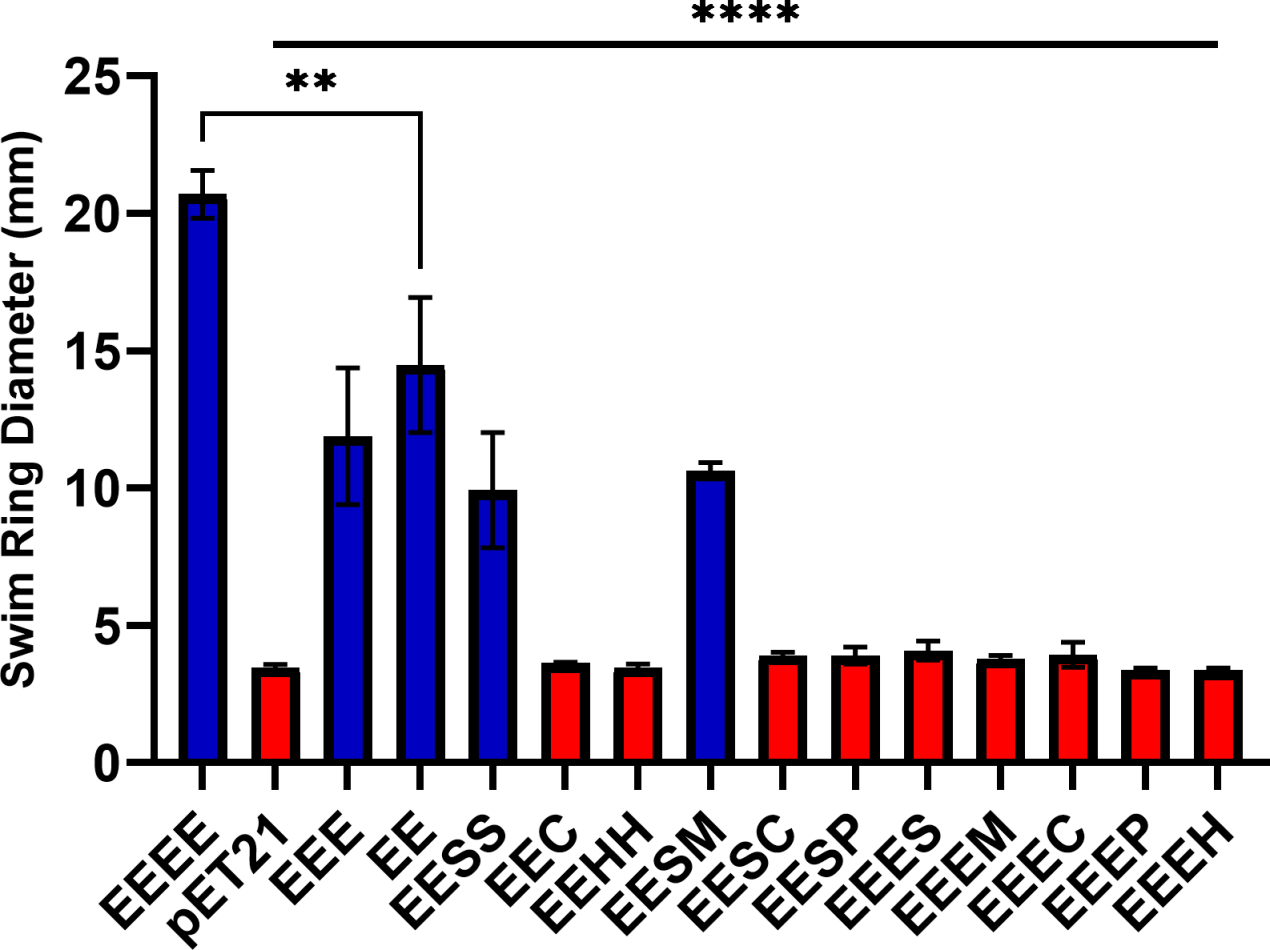
Mean swim ring diameter of cells expressing each chimeric and outer domain-deleted FliC recorded from three independent 0.3% swim plates incubated at 30°C for 16 h. Motile and non-motile flagellin variants expressing chimeric FliC are indicated by blue and red bars, respectively. Error bars indicate standard error of the mean. A two-way analysis of variance comparing each column to the FliC value was used to generate the *P* values. **P ≤ 0.01, ***P ≤0.001.

Next, we investigated whether chimeric FliC could form functional filaments and therefore rescue motility in filament-disrupted strains (*fliC::tetRA*). Plasmids expressing FliC mutants or flagellar stators were co-transformed into an *E. coli* Δ*motAB fliC::tetRA* strain. Motility of outer domain-deleted and chimeric FliC-expressing strains was confirmed using swim plate motility assays. Both outer domain deletions produced swim rings (Fig. 3). The average swim ring diameter when both outer domains were deleted (EE) was larger, 14.5 ± 2.5 mm, than that with only the D3 domain deleted, 11.9 ± 2.5 mm; however, this difference was not statistically significant (Fig. 3). However, both these outer domain-deleted flagellin mutants produced significantly smaller swim rings than wild-type FliC. Likewise, motility of the two-species outer domain FliC mutants, EESS, EEH, and EEC, was confirmed using a swim plate motility assay. Only EESS was motile, with a swim ring diameter of 9.9 ± 2.1 mm (Fig. 3). EEC and EEH produced no swim rings (Fig. 3). Only one of the three-species chimeras was observed to be motile. The FliC chimera that contained the D3 domain from *Mesorhizobium* sp. OR3359 consistently produced a swim ring diameter of 10.6 ± 0.3 mm, whereas EESC and EESP were not motile (Fig. 3). All flagellin chimeras with the D3 domain of *E. coli* K-12 replaced by the outermost domain of another bacterial flagellin were non-motile.

As the viscosity of agar plates was increased, the swim ring diameter decreased significantly (Fig. S5A), with the overall relationship between the chimeric FliC-expressing strains and WT FliC remaining the same (Fig. S5A). However, when normalized to the swim ring diameters using 0.3% agar, there were no significant differences in the percentage of the swim ring diameter between any motile flagellin variant and wild type at either 0.35% or 0.4% agar (Fig. S5B).

### Motile FliC chimeras that produced significantly lower swim speeds

**Fig 4:**
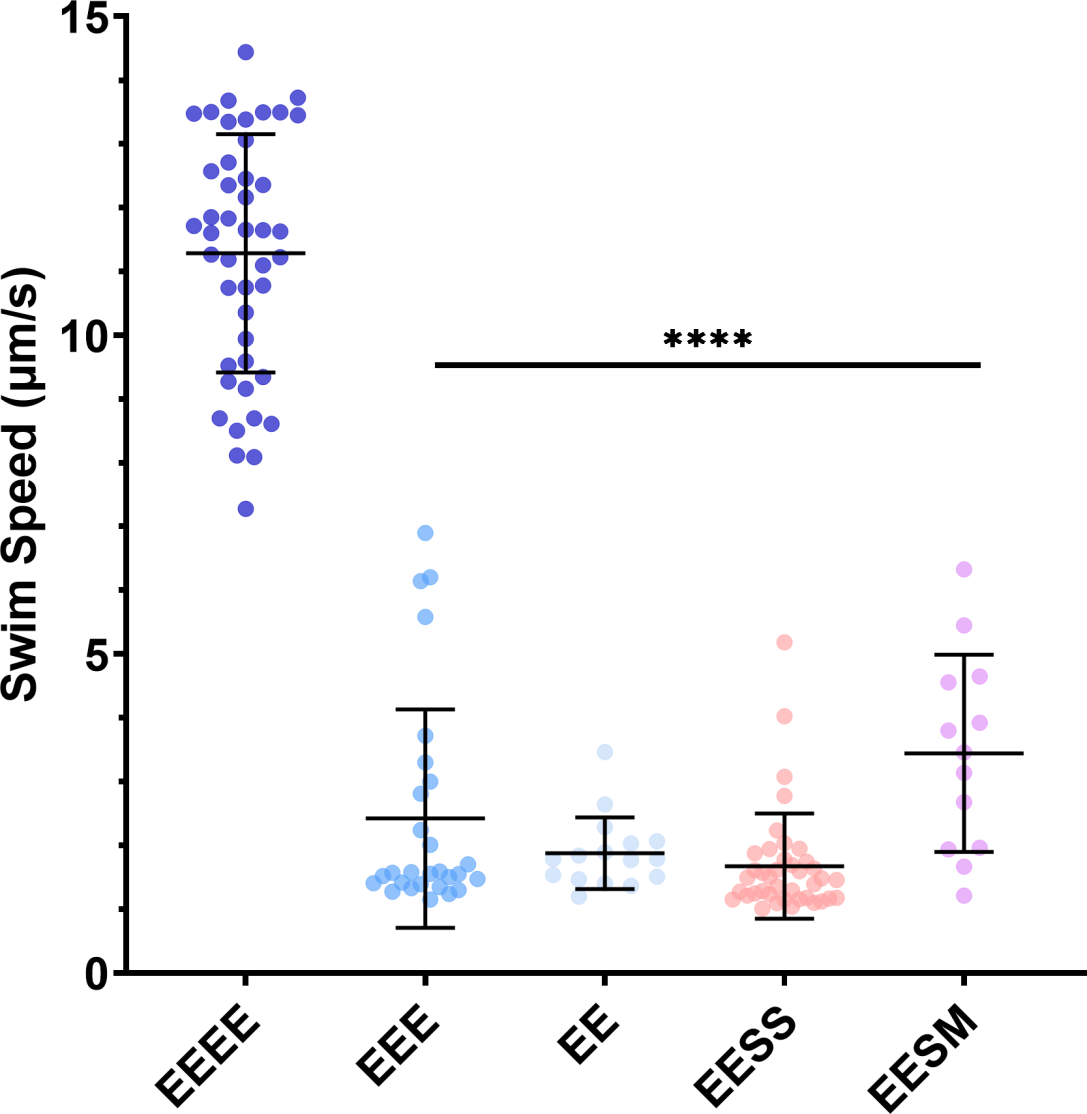
Free-swimming speeds for motile chimeric and truncated flagellin (in µm/s). Error bars indicate standard deviation. A two-way analysis of variance comparing each column to EEEE was used to generate the *P* values. Cells expressing wild-type FliC (EEEE) are significantly different from all other FliC variants (*P* value ≤ 0.05). ****P ≤ 0.0001.

Free swimming assays were used to measure the swimming speeds for each of the motile FliC strains that were generated as part of this work. Strains expressing EEEE (11.2 ± 2.3 µm/s) had swim speeds significantly faster than the other motile variants (*P* value of <0.0001) (Fig. 4). Strains expressing EEE (2.4 ± 1.7 µm/s), EE (1.9 ± 0.6 µm/s), EESS (1.7 ± 0.8 µm/s), and EESM (3.44 ± 1.5 µm/s) had significantly lower swim speeds.

### Crystal violet staining of filaments shows formation of chimeric filaments

**Fig 5:**
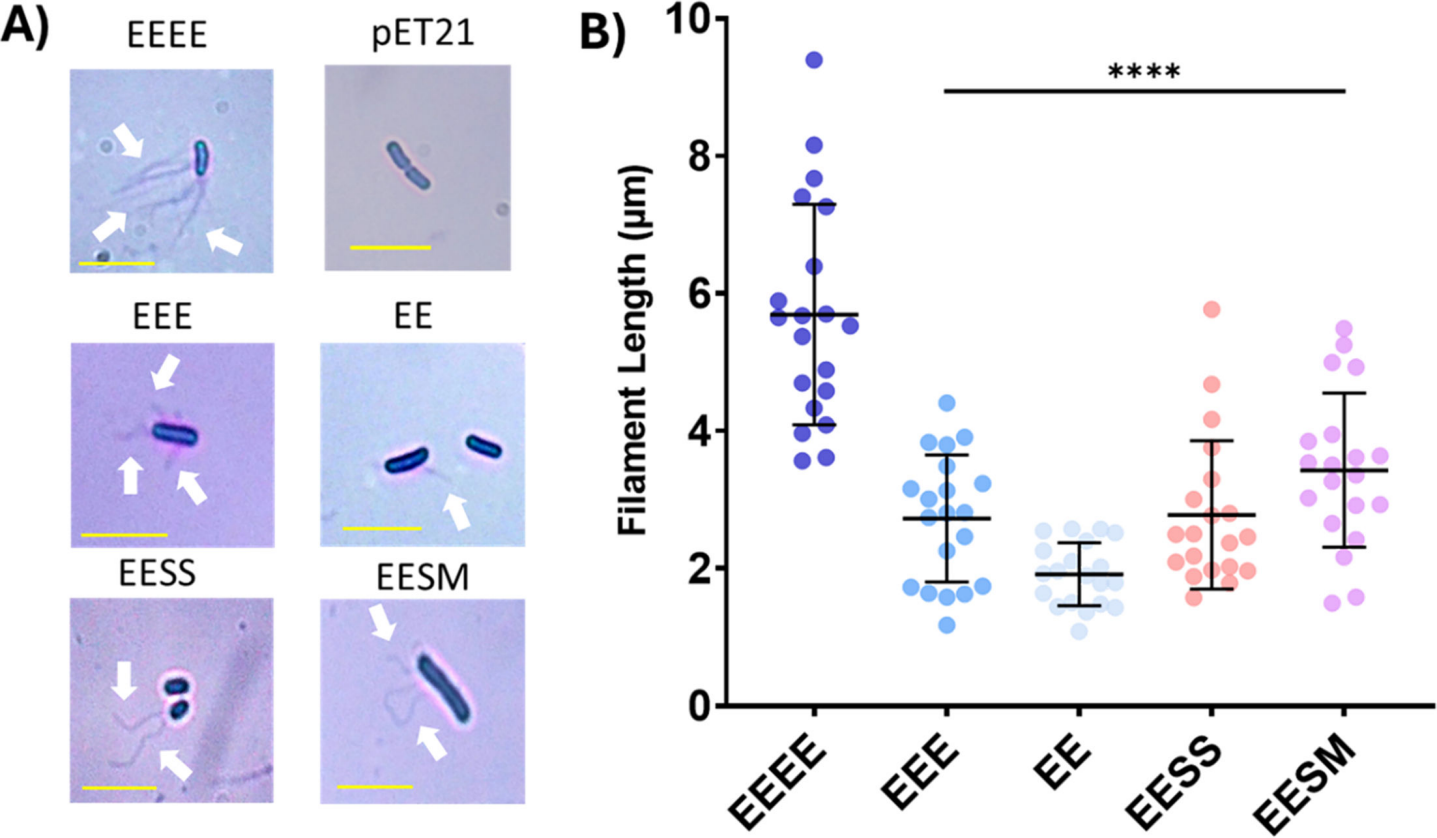
**(A)** Analysis of filament export and assembly. Representative images of motile strains after flagella staining. The pET21 empty vector is shown as a representative non-motile variant. Scale bar (yellow) represents 5 µm. White arrows indicate the presence of flagella. (**B**) Lengths of FliC chimeric filaments. Error bars represent standard deviation. A two-way analysis of variance comparing each column to FliC was used to generate the *P* values. ****P ≤ 0.0001.

Staining of flagella confirmed export of flagellin and the formation of filaments. Filaments were observed in all motile strains (Fig. 5A). For all non-motile strains, there were no observable filaments. Filament length was determined for all motile strains, with EEEE (FliC) producing the longest filaments at 5.7 ± 1.6 µm (Fig. 5B). The outer domain-deleted constructs EEE and EE produced significantly shorter filaments at 2.7 ± 0.9 µm and 1.9 ± 0.5 µm, respectively. Shorter filaments were also formed in the two-species chimera EESS (2.8 ± 1.1 µm) and in the three-species chimera EESM (3.4 ± 1.1 µm) (Fig. 5B). All motile cells expressing domain-deleted or chimeric FliC had significantly shorter filaments than cells expressing wild-type FliC (EEEE).

## DISCUSSION

In this study, we investigated whether expression of *E. coli* FliC with deleted or replaced outer domains (D2 and D3) could rescue motility in *fliC*-deleted strains of *E. coli*. We observed that flagellin outer domains were not required for motility in *E. coli* and that two- and three-species FliC outer domain chimeras are able to form functional filaments. From our phylogenetic analysis, we showed that bacteria have evolved flagellar filaments with and without outer domains. The presence or absence of these domains is distributed in a way that correlates with bacterial clades. A greater proportion of flagellins with outer domains was isolated from animal sources. Previous work has shown that the presence of these hypervariable domains can aid in immune evasion (42). Horizontal gene transfer of complete sets of flagellar genes has been demonstrated as well as evidence of specific recombination of the flagellin outer domains (19, 43, 44). Here, we constructed FliC with chimeric or deleted outer domains to show that recombination of outer domains can form functional filaments, thus providing evidence that recombination events can occur across multiple genera with compatible outer domains.

### FliC outer domains can affect bacterial motility

Although originally thought that the outer domains have no interaction with the core domains or each other (7, 13), recent studies have shown that in some cases, there are inter-domain interactions which help to stabilize the filament to aid motility (21). In Kreutzberger et al., this phenomenon was identified in *Achromobacter* filaments where the outer domains exist in four conformations and can form a tetrameric sheath which surrounds the filament (22). The authors also observed outer domain interactions in *Sinorhizobium meliloti* forming a dimeric outer domain interaction in two conformations (22). Similarly, *P. aeruginosa* PAO1 flagellin has been shown to form a ridged filament due to interactions of its outer domains (21). This suggests that, along with the well-studied role of immune activation of the flagellin outer domains, there is also a role that these domains have on bacterial swim speed in different environments (25). In *S*. Typhimurium and *E. coli*, it has been previously shown that partial outer domain-deleted FliC mutants are motile (45, 46); however, full D2/D3 deletions were non-motile, whereas our results show that *E. coli* K-12 expressing FliC deleted for domains D2 and D3 is still motile to some degree. This demonstrates that the outer domains of the *E. coli* K-12 flagellin are not absolutely required for motility. We observed rescue in motility when replacing the D2/D3 domains with those from the *S*. Typhimurium flagellin as well as a two-species chimera with *S*. Typhimurium and *Mesorhizobium* sp. ORS 3359. Future structural work would characterize outer domain interactions in these mutant filaments and quantify any effect on the superstructure of the filament.

The lack of motility in the majority of chimeric flagellin-expressing strains could also arise from the lack of post-translational modifications. It is known that many flagellins, although not *E. coli* K-12, require glycosylation or methylation for the formation of the flagella filament (47, 48). *Epsilonproteobacteria*, in which *H. mustelae* is found, require glycosylation for the formation of the flagellar filament (49). Both *P. aeruginosa* a-type and b-type flagellins also are glycosylated on their exposed surfaces (50). This may be the reason why no motility of some of the flagellin chimera-expressing strains was observed. The levels of flagellin expression may also be an issue, compounded with complex flagellin structures that could potentially influence the formation of the flagellar filament. Previous work showed that motility was only partially restored (~50%) when a rhamnose promoter was used when compared to the wild-type flagellin promoter (51). Therefore, optimization of the flagellin expression system, using various promoters and/or expression vectors, could increase the levels of chimeric flagellin expression and thus increase the likelihood of the formation of the flagellar filaments. Additionally, due to the use of multiple antibiotics to select and maintain the two expression plasmids, there may have been a reduction in flagellin expression and therefore a reduced likelihood of functional filament formation (52).

Bacterial swim speed can vary widely, with *E. coli* swim speeds ranging from 14.2 to 28.8 µm/s, *Bdellovibrio bacteriovorus* at 100 µm/s, and *Thiovulum majus* at approximately 600 µm/s (53–56). *E. coli* and *T. majus* both contain flagellin outer domains, whereas the *B. bacteriovorus* flagellin does not. We observed lower swim speeds and shorter filaments for EESS, EESM, EEE, and EE expressing bacteria. Previous work has shown that even a single polymorphism (at position N87 in *E. coli* K-12 FliC) prevented motility in structured environments, although motility was observed in liquids (29). Therefore, it is plausible that whole domain replacements may alter the polymorphic transformation of the flagellar filament, which allows for raised observable motility within soft agar. Additionally, reductions in motility may arise due to weaker filaments being more prone to shearing or due to differences in the filament export rate.

### Outer domain chimeras require *S*. *Typhimurium* D2 domain to form filaments

We observed that 2 out of 11 FliC variants rescued motility, with the remainder of the chimeras being non-motile. In work by Nedeljkocić et al., 13 flagellin chimeras, with combinations of all domains, were generated using the FliC from *S*. *Typhimurium* and *P. aeruginosa* PAO1, and only 1 multi-genus chimera produced a functional filament (21). Furthermore, in the same study, the authors generated flagellin chimeras using domain swaps of two *P. aeruginosa* strains (PAO1 and PAK), where only two variants (where the D0 domain was exchanged) were motile (21). This further illustrates that the production of flagellin chimeras even within the same bacterial species does not yield a high proportion of motile variants. In *E. coli* SYC29 expressing chimeric-FliC containing outer domains, motility was only rescued in chimeras that contained the *S*. Typhimurium D2 domain. This may indicate that there are key structural elements or residues found in this domain that allow export and formation of a functional filament.

### Conclusion

Engineering of the flagellin outer domains can not only increase our understanding of filament formation but also could provide biotechnological benefits. Flagellin is already used to display antigens from other organisms and stimulate the immune system and has been engineered to contain fluorescent and enzymatic domains (25, 57, 58). Here we sought to develop chimeras for the outer domains of *E. coli* K-12 flagellin to provide evidence that recombination events have the potential to produce functional filaments. We have shown that two- and three-species chimeras can form functional filaments. However, our work illustrates the challenges of exchanging whole flagellin outer domains between bacteria. Further analysis is required to fully understand the export, folding, and incorporation of chimeric flagellins and how they interact with other proteins within the bacterial flagellar motor. Future work will investigate how hypervariable FliC domains can affect motility in response to environmental changes and establish FliC and its homologs as a synthetic biology platform for biotechnological applications.

## SUPPLEMENTAL MATERIAL

Phylogeny S1 jb.00517-24-s0001.txt Nexus file for phylogeny in Fig. 1.

Phylogeny S2 jb.00517-24-s0002.txt Nexus file for phylogeny in Fig. S1.

Supplemental material jb.00517-24-s0003.pdf Tables S1 to S5, Fig. S1 to S5, and legends for phylogenies S1 and S2.

ASM does not own the copyrights to Supplemental Material that may be linked to, or accessed through, an article. The authors have granted ASM a non-exclusive, world-wide license to publish the Supplemental Material files. Please contact the corresponding author directly for reuse.
