# Supplementary material for: Rescue of bacterial motility using two- and three-species FliC chimeras
Source: J Bacteriol. 2025 Aug 11;207(9):e00517-24. doi: 10.1128/jb.00517-24 (PMC12445082; doi:10.1128/jb.00517-24)
Supplement: Supplemental material — Tables S1 to S5, Fig. S1 to S5, and legends for phylogenies S1 and S2. [file jb.00517-24-s0003.pdf]

Supplementary Material: Rescue of bacterial motility using two and three-species FliC chimeras

Tables S1-5

Figures S1-S5

Jacob Scadden[1], Pietro Ridone[1], Divyangi Pandit[1], Yoshiyuki Sowa[2,3], Matthew AB Baker[1*]

[1]School of Biotechnology and Biomolecular Sciences, University of New South Wales, Sydney, Australia
[2]Department of Frontier Bioscience, Hosei University, Tokyo, Japan
[3]Research Center for Micro-Nano Technology, Hosei University, Tokyo, Japan

*Corresponding Author

Table S1. Bacterial strains and plasmids used in this study.

**Strain**	**Relevant Characteristics**	**Reference**
E. coli NEB 10-β	DH10B derivative. Shuttle vector cloning strain	New England Biolabs (C3019)
E. coli SYC29	*E. coli* RP437, Δ*motAB fliC*::*tetRA*, TET^R	This study
**Plasmid**	**Relevant Characteristics**	**Reference**
pET21(+)	Expression vector, T7 promoter, Amp^R, IPTG induction	Merck (69740)
pDB108	pBAD33 backbone, motAB, CAM^R, arabinose induction	David Blair* (University of Utah, USA)
pET21_*EEEE*	Wild type E. coli *fliC*, pET21a backbone	This study
pET21_*EEE*	*E. coli fliC* with deletion of 195-300, pET21a backbone	This study
pET21_*EE*	*fliC* with deletion of 174-405, pET21a backbone	This study
pET21_*EESS*	*E. coli fliC* with D2/3 domains replaced by *Salmonella* Typhimurium strain LT2 D2/3 domains, pET21a backbone	This study
pET21_*EESM*	*E. coli fliC* with D2/3 domains replaced by *Salmonella* Typhimurium strain LT2 D2 domain and the *Mesorhizobium* sp. ORS 3359 D2 domain, pET21a backbone	This study
pET21_*EESP*	*E. coli fliC* with D2/3 domains replaced by *Salmonella* Typhimurium strain LT2 D2 domain and the *Pseudomonas aeruginosa* D3 domain, pET21a backbone	This study
pET21_*EESC*	*E. coli fliC* with D2/3 domains replaced by *Salmonella*	This study

	Typhimurium strain LT2 D2 domain and the *Collimonas fungivorans* strain Ter331 D2 domain, pET21a backbone	
pET21_*EEHH*	*E. coli fliC* with D2/3 domains replaced by the *Helicobacter mustelae* D2/3 domains, pET21a backbone	This study
pET21_*EEC*	*E. coli fliC* with D2/3 domains replaced by the *Collimonas fungivorans* strain Ter331 D2 domains, pET21a backbone	This study
pET21_EEES	*E. coli fliC* with the D3 domain replaced by the *Salmonella* Typhimurium strain LT2 D3 domain, pET21a backbone	This Study
pET21_EEEM	*E. coli fliC* with the D3 domain replaced by the *Mesorhizobium* sp. ORS 3359 D2 domain, pET21a backbone	This Study
pET21_EEEC	*E. coli fliC* with the D3 domain replaced by the *Collimonas fungivorans* strain Ter331 D2 domain, pET21a backbone	This Study
pET21_EEEP	*E. coli fliC* with the D3 domain replaced by the *Pseudomonas aeruginosa* D3 domain, pET21a backbone	This Study
pET21_EEEH	*E. coli fliC* with the D3 domain replaced by the *Helicobacter mustelae* D3 domain, pET21a backbone	This Study

Kindly gifted from the *Blair Lab

Table S2. Flagellin reference sequences used in the BLASTP search. The final row, FliD, (**Bold**) was used as the outgroup (P24216).

**UniProt ID**	**Bacteria**	**Protein ID**	**Length (Amino Acids)**	**D2/3 Domains Present**
P04949	*E. coli* (strain K12)	Flagellin	498	Yes
A0A1H7N2K9	*Blastococcus* sp. DSM 46786	Flagellin	277	No
P13118	*Rhizobium meliloti*	Flagellin A	395	D2 Only
Q06064	*Bordetella bronchiseptica* (strain ATCC BAA-588)	Flagellin	391	D2 Only
Q6AJQ8	*Desulfotalea psychrophile* (DSM 12343)	Flagellin	857	Yes
P0A0S1	*Helicobacter pylori* (strain ATCC 700392)	Flagellin A	510	Yes
P80583	*Clostridium tyrobutyricum*	Flagellin	377	D2 Only

A0A1N6WG11	*Alkalispirochaeta americana*	Flagellin	284	No
**P24216**	***E. coli* (strain K12)**	**Flagellar hook-associated protein**	**468**	**N/A**

Table S3. Parameters used for the generation of the flagellin phylogeny using IQTree on ACCESS.

IQ-Tree on ACCESS - Parameters	
abayes_test_	FALSE
datatype_	PROTEIN
extract_datefile_	FALSE
fix_branchlengths_	FALSE
lbp_test_	FALSE
median_approximation_	FALSE
more_memory2_	FALSE
more_memory_	FALSE
no_mlpairwise_	FALSE
optimize_weights_	FALSE
parametrical_test_	FALSE
per_sitefile_	FALSE
per_sitewplfile_	FALSE
per_sitewslfile_	FALSE
per_sitewslmfile_	FALSE
per_sitewslmrfile_	FALSE
per_sitewslrfile_	FALSE
per_sitewspmfile_	FALSE
per_sitewspmrfile_	FALSE
per_sitewsprfile_	FALSE
print_sitestats_	FALSE
runtime_	0.5
sh_test_	FALSE
slower_NNI_	FALSE
specify_fulltreesearch_	FALSE
specify_numpatterns_	921
specify_prefix_	output
specify_radius_	6
specify_safe_	FALSE
specify_symtype_	pval
thorough_estimation_	FALSE
unbiased_test_	FALSE
use_fasttreesearch_	FALSE
use_nj_	FALSE
use_random_	FALSE
use_symmtest_	FALSE

which_iqtree_	232
write_ancestralseqs_	FALSE
write_locally_optimal_	FALSE
write_loglikelihoods_	FALSE
write_sitelikelihoods_	FALSE

Table S4. Sequences of FliC chimeras and outer domain deleted FliC. Sequences are coloured based on domains incorporated into the outer domains for *E. coli* K-12 FliC.

FliC Variant	Domains Present	Amino Acid Sequence	Length
EEEE	D0, D1, D2, D3	MAQVINTNSLSLITQNNINKNQSALSSSIERLSSGLRINSAKDDAAGQAIANRFTSNIKGLTQ<br>AARNANDGISVAQTTEGALSEINNNLQRVRELTVQATTGTNSESDLSSIQDEIKSRLDEIDRV<br>SGQTQFNGVNVLAKNGSMKIQVGANDNQTITIDLKQIDAKTLGLDGFSVKNNDTVTTSAPV<br>TAFGATTTNNIKLTGITLSTEAATDTGGTNPASIEGVYTDNGNDYYAKITGGDNDGKYYAVTV<br>ANDGTVTMATGATANATVTDANTTKATTITSGGTPVQIDNTAGSATANLGAVSLVKLQDSKG<br>NDTDTYALKDTNGNLYAADVNETTGAVSVKTITYTDSSGAASSPTAVKLGGDDGKTEVVDID<br>GKTYDSADLNGGNLQTGLTAGGEALTAVANGKTTDPLKALDDAIASVDKFRSSLGAVQNRL<br>DSAVTNLNNTTTNLSEAQSRIQDADYATEVSNMSKAQIIQQAGNSVLAKANQVPQQVLSLL<br>QG	498
EEE	D0, D1, D2	MAQVINTNSLSLITQNNINKNQSALSSSIERLSSGLRINSAKDDAAGQAIANRFTSNIKGLTQ<br>AARNANDGISVAQTTEGALSEINNNLQRVRELTVQATTGTNSESDLSSIQDEIKSRLDEIDRV<br>SGQTQFNGVNVLAKNGSMKIQVGANDNQTITIDLKQIDAKTLGLDGFSVKNNDTVTTSAPV<br>TAFGATTAVSLVKLQDSKGNDTDTYALKDTNGNLYAADVNETTGAVSVKTITYTDSSGAASS<br>PTAVKLGGDDGKTEVVDIDGKTYDSADLNGGNLQTGLTAGGEALTAVANGKTTDPLKALDD<br>AIASVDKFRSSLGAVQNRLDSAVTNLNNTTTNLSEAQSRIQDADYATEVSNMSKAQIIQQAG<br>NSVLAKANQVPQQVLSLLQG	393
EE	D0, D1	MAQVINTNSLSLITQNNINKNQSALSSSIERLSSGLRINSAKDDAAGQAIANRFTSNIKGLTQ<br>AARNANDGISVAQTTEGALSEINNNLQRVRELTVQATTGTNSESDLSSIQDEIKSRLDEIDRV<br>SGQTQFNGVNVLAKNGSMKIQVGANDNQTITIDLKQIDAKTLGLDGFKTTDPLKALDDAIAS<br>VDKFRSSLGAVQNRLDSAVTNLNNTTTNLSEAQSRIQDADYATEVSNMSKAQIIQQAGNSV<br>LAKANQVPQQVLSLLQG	266
EEHH	D0, D1, D2, D3	MAQVINTNSLSLITQNNINKNQSALSSSIERLSSGLRINSAKDDAAGQAIANRFTSNIKGLTQ<br>AARNANDGISVAQTTEGALSEINNNLQRVRELTVQATTGTNSESDLSSIQDEIKSRLDEIDRV<br>SGQTQFNGVNVLAKNGSMKIQVGANDNQTITIDLKQIDAKTLGLDGFQVRINTGAMITAAS<br>EATLTFKQINGGGTSPLEGVKISHSVGTGLGVLAEVINKNSDKTGIRAKASVETTSDKEIMSG<br>NLKNLTINDVNIGNIVDIKKGDADGRLVQAINALTSSTGVEASTDSKGRLNLRSVDGRGIVLK<br>ADASEDNGDGKSAPMAIDAVNGGQSITDGEGAANYGRLSLVRLDARDIVLTSSDKPDENK<br>FSAIGFGDNNVAMATVNLRDVLGKFDASVKSASGANYNAVIASGNSNLKTTDPLKALDDAI<br>ASVDKFRSSLGAVQNRLDSAVTNLNNTTTNLSEAQSRIQDADYATEVSNMSKAQIIQQAGN<br>SVLAKANQVPQQVLSLLQG	514
EEC	D0, D1, D2	MAQVINTNSLSLITQNNINKNQSALSSSIERLSSGLRINSAKDDAAGQAIANRFTSNIKGLTQ<br>AARNANDGISVAQTTEGALSEINNNLQRVRELTVQATTGTNSESDLSSIQDEIKSRLDEIDRV<br>SGQTQFNGVNVLAKNGSMKIQVGANDNQTITIDLKQIDAKTLGLDGFNTLNLAGPSGATAA<br>MTPTGKDNTGASVAVAAGGFTAVNRDAAAGFTATVADLVTDARGNTYVKNGANFYAATVT<br>VADATQGATAATPTATVTYDSSKSLTAASGSPLKTTDPLKALDDAIASVDKFRSSLGAVQNRL<br>DSAVTNLNNTTTNLSEAQSRIQDADYATEVSNMSKAQIIQQAGNSVLAKANQVPQQVLSLL<br>QG	373
EESS	D0, D1, D2, D3	MAQVINTNSLSLITQNNINKNQSALSSSIERLSSGLRINSAKDDAAGQAIANRFTSNIKGLTQ<br>AARNANDGISVAQTTEGALSEINNNLQRVRELTVQATTGTNSESDLSSIQDEIKSRLDEIDRV<br>SGQTQFNGVNVLAKNGSMKIQVGANDNQTITIDLKQIDAKTLGLDGFQKYKVSDTAATVTG	499

		YADTTIALDNSTFKASATGLGGTDQKIDGDLKFDDTTGKYYAKVTVTGGTGKDGYYEVSVDKTNGEVTLAGGATSPLTGGLPATATEDVKNVQVANADLTEAKAALTAAGVTGTASVVKMSYTDNNGKTIDGGLAVKVGDDYYSATQNKDGSISINTTKYTADDGTSKTALNKLGGADGKTEVVSIGGKTYAASKAEGHNFKAQPDLAEAAATTTENPLKTTDPLKALDDAIASVDKFRSSLGAVQNRLDSAVTNLNNTTTNLSEAQSRIQDADYATEVSNMSKAQIIQQAGNSVLAKANQVPQQVLSLLQG	
EESM	D0, D1, D2, D2	MAQVINTNSLSLITQNNINKNQSALSSSIERLSSGLRINSAKDDAAGQAIANRFTSNIKGLTQAARNANDGISVAQTTEGALSEINNNLQRVRELTVQATTGTNSESDLSSIQDEIKSRLDEIDRVSGQTQFNGVNVLAKNGSMKIQVGANDNQTITIDLKQIDAKTLGLDGFQKYKVSDTAATVTINVESTKLFDSGLSTAVKNQGILDRKTSIYSTAADQSAYDAAYATAIAGGATDIAANTAGQAAVAGTPVKIDNVSAFNLDITATGVTDDIITQMVTNADLTEAKAALTAAGVTGTASVVKMSYTDNNGKTIDGGLAVKVGDDYYSATQNKDGSISINTTKYTADDGTSKTALNKLGGADGKTEVVSIGGKTYAASKAEGHNFKAQPDLAEAAATTTENPLKTTDPLKALDDAIASVDKFRSSLGAVQNRLDSAVTNLNNTTTNLSEAQSRIQDADYATEVSNMSKAQIIQQAGNSVLAKANQVPQQVLSLLQG	499
EESC	D0, D1, D2, D2	MAQVINTNSLSLITQNNINKNQSALSSSIERLSSGLRINSAKDDAAGQAIANRFTSNIKGLTQAARNANDGISVAQTTEGALSEINNNLQRVRELTVQATTGTNSESDLSSIQDEIKSRLDEIDRVSGQTQFNGVNVLAKNGSMKIQVGANDNQTITIDLKQIDAKTLGLDGFQKYKVSDTAATVTNTLNLAGPSGATAAMTPTGKDNTGASVAVAAGGFTAVNRDAAAGFTATVADLVTDARGNTYVKNGANFYAATVTVADATQGATAATPTATVTYDSSKSLTAASGSPLNADLTEAKAALTAAGVTGTASVVKMSYTDNNGKTIDGGLAVKVGDDYYSATQNKDGSISINTTKYTADDGTSKTALNKLGGADGKTEVVSIGGKTYAASKAEGHNFKAQPDLAEAAATTTENPLKTTDPLKALDDAIASVDKFRSSLGAVQNRLDSAVTNLNNTTTNLSEAQSRIQDADYATEVSNMSKAQIIQQAGNSVLAKANQVPQQVLSLLQG	510
EESP	D0, D1, D2, D2	MAQVINTNSLSLITQNNINKNQSALSSSIERLSSGLRINSAKDDAAGQAIANRFTSNIKGLTQAARNANDGISVAQTTEGALSEINNNLQRVRELTVQATTGTNSESDLSSIQDEIKSRLDEIDRVSGQTQFNGVNVLAKNGSMKIQVGANDNQTITIDLKQIDAKTLGLDGFQKYKVSDTAATVTNGTYFKADGGGAVTAATASGTVDIAIGITGGSAVNVKVDMKGNETAEQAAAKIAAAVNDANVGIGAFSDGDTISYVSKAGKDGSGAITSAVSGVVIADTGSTGVGTAAGVTPSATAFAKTNDTVAKIDISTANADLTEAKAALTAAGVTGTASVVKMSYTDNNGKTIDGGLAVKVGDDYYSATQNKDGSISINTTKYTADDGTSKTALNKLGGADGKTEVVSIGGKTYAASKAEGHNFKAQPDLAEAAATTTENPLKTTDPLKALDDAIASVDKFRSSLGAVQNRLDSAVTNLNNTTTNLSEAQSRIQDADYATEVSNMSKAQIIQQAGNSVLAKANQVPQQVLSLLQG	535
EEES	D0, D1, D2, D3	MAQVINTNSLSLITQNNINKNQSALSSSIERLSSGLRINSAKDDAAGQAIANRFTSNIKGLTQAARNANDGISVAQTTEGALSEINNNLQRVRELTVQATTGTNSESDLSSIQDEIKSRLDEIDRVSGQTQFNGVNVLAKNGSMKIQVGANDNQTITIDLKQIDAKTLGLDGFSVKNNDTVTTSAPVTGYADTTIALDNSTFKASATGLGGTDQKIDGDLKFDDTTGKYYAKVTVTGGTGKDGYYEVSVDKTNGEVTLAGGATSPLTGGLPATATEDVKNVQVAAFGATTAVSLVKLQDSKGNDTDTYALKDTNGNLYAADVNETTGAVSVKTITYTDSSGAASSPTAVKLGGDDGKTEVVDIDGKTYDSADLNGGNLQTGLTAGGEALTAVANGKTTDPLKALDDAIASVDKFRSSLGAVQNRLDSAVTNLNNTTTNLSEAQSRIQDADYATEVSNMSKAQIIQQAGNSVLAKANQVPQQVLSLLQG	488
EEEM	D0, D1, D2, D2	MAQVINTNSLSLITQNNINKNQSALSSSIERLSSGLRINSAKDDAAGQAIANRFTSNIKGLTQAARNANDGISVAQTTEGALSEINNNLQRVRELTVQATTGTNSESDLSSIQDEIKSRLDEIDRVSGQTQFNGVNVLAKNGSMKIQVGANDNQTITIDLKQIDAKTLGLDGFSVKNNDTVTTSAPVTINVESTKLFDSGLSTAVKNQGILDRKTSIYSTAADQSAYDAAYATAIAGGATDIAANTAGQAAVAGTPVKIDNVSAFNLDITATGVTDDIITQMVTAFGATTAVSLVKLQDSKGNDTDTYALKDTNGNLYAADVNETTGAVSVKTITYTDSSGAASSPTAVKLGGDDGKTEVVDIDGKTYDSADLNGGNLQTGLTAGGEALTAVANGKTTDPLKALDDAIASVDKFRSSLGAVQNRLDSAVTNLNNTTTNLSEAQSRIQDADYATEVSNMSKAQIIQQAGNSVLAKANQVPQQVLSLLQG	488
EEEC	D0, D1, D2, D2	MAQVINTNSLSLITQNNINKNQSALSSSIERLSSGLRINSAKDDAAGQAIANRFTSNIKGLTQAARNANDGISVAQTTEGALSEINNNLQRVRELTVQATTGTNSESDLSSIQDEIKSRLDEIDRVSGQTQFNGVNVLAKNGSMKIQVGANDNQTITIDLKQIDAKTLGLDGFSVKNNDTVTTSAPV	489

		TNTLNLAGPSGATAAMTPTGKDNTGASVAVAAGGFTAVNRDAAAGFTATVADLVTDARGNTYVKNGANFYAATVTVADATQGATAATPTATVTYDSSKSLTAASGSPLAFGATTAVSLVKLQDSKGNDTDTYALKDTNGNLYAADVNETTGAVSVKTITYTDSSGAASSPTAVKLGGDDGKTEVVDIDGKTYDSADLNGGNLQTGLTAGGEALTAVANGKTTDPLKALDDAIASVDKFRSSLGAVQNRLDSAVTNLNNTTTNLSEAQSRIQDADYATEVSNMSKAQIIQQAGNSVLAKANQVPQQVLSLLQG	
EEEP	D0, D1, D2, D3	MAQVINTNSLSLITQNNINKNQSALSSSIERLSSGLRINSAKDDAAGQAIANRFTSNIKGLTQAARNANDGISVAQTTEGALSEINNNLQRVRELTVQATTGTNSESDLSSIQDEIKSRLDEIDRVSGQTQFNGVNVLAKNGSMKIQVGANDNQTITIDLKQIDAKTLGLDGFSVKNNDTVTTSAPVTNGTYFKADGGGAVTAATASGTVDIAIGITGGSAVNVKVDMKGNETAEQAAAKIAAAVNDANVGIGAFSDGDTISYVSKAGKDGSGAITSAVSGVVIADTGSTGVGTAAGVTPSATAFAKTNDTVAKIDISTAAFGATTAVSLVKLQDSKGNDTDTYALKDTNGNLYAADVNETTGAVSVKTITYTDSSGAASSPTAVKLGGDDGKTEVVDIDGKTYDSADLNGGNLQTGLTAGGEALTAVANGKTTDPLKALDDAIASVDKFRSSLGAVQNRLDSAVTNLNNTTTNLSEAQSRIQDADYATEVSNMSKAQIIQQAGNSVLAKANQVPQQVLSLLQG	522
EEEH	D0, D1, D2, D3	MAQVINTNSLSLITQNNINKNQSALSSSIERLSSGLRINSAKDDAAGQAIANRFTSNIKGLTQAARNANDGISVAQTTEGALSEINNNLQRVRELTVQATTGTNSESDLSSIQDEIKSRLDEIDRVSGQTQFNGVNVLAKNGSMKIQVGANDNQTITIDLKQIDAKTLGLDGFSVKNNDTVTTSAPVTDKEIMSGNLKNLTINDVNIGNIVDIKKGDADGRLVQAINALTSSTGVEASTDSKGRLNLRSVDGRGIVLKADASEDNGDGKSAPMAIDAVNGGQSITDGEGAAFGATTAVSLVKLQDSKGNDTDTYALKDTNGNLYAADVNETTGAVSVKTITYTDSSGAASSPTAVKLGGDDGKTEVVDIDGKTYDSADLNGGNLQTGLTAGGEALTAVANGKTTDPLKALDDAIASVDKFRSSLGAVQNRLDSAVTNLNNTTTNLSEAQSRIQDADYATEVSNMSKAQIIQQAGNSVLAKANQVPQQVLSLLQG	492

Table S5. Primers and PCR conditions used in this study to confirm chimeric and outer domain deleted *fliC* into pET21(+).

Primer Name	Template Sequence	5' -> 3' Sequence	Tm (°C)	Length
pET21_F	pET21(+)	TTCCTTTCGGGCTTTGTTAGCAG	66.4	23
pET21_R	pET21(+)	GGATCGAGATCTCGATCCCGC	66.9	21

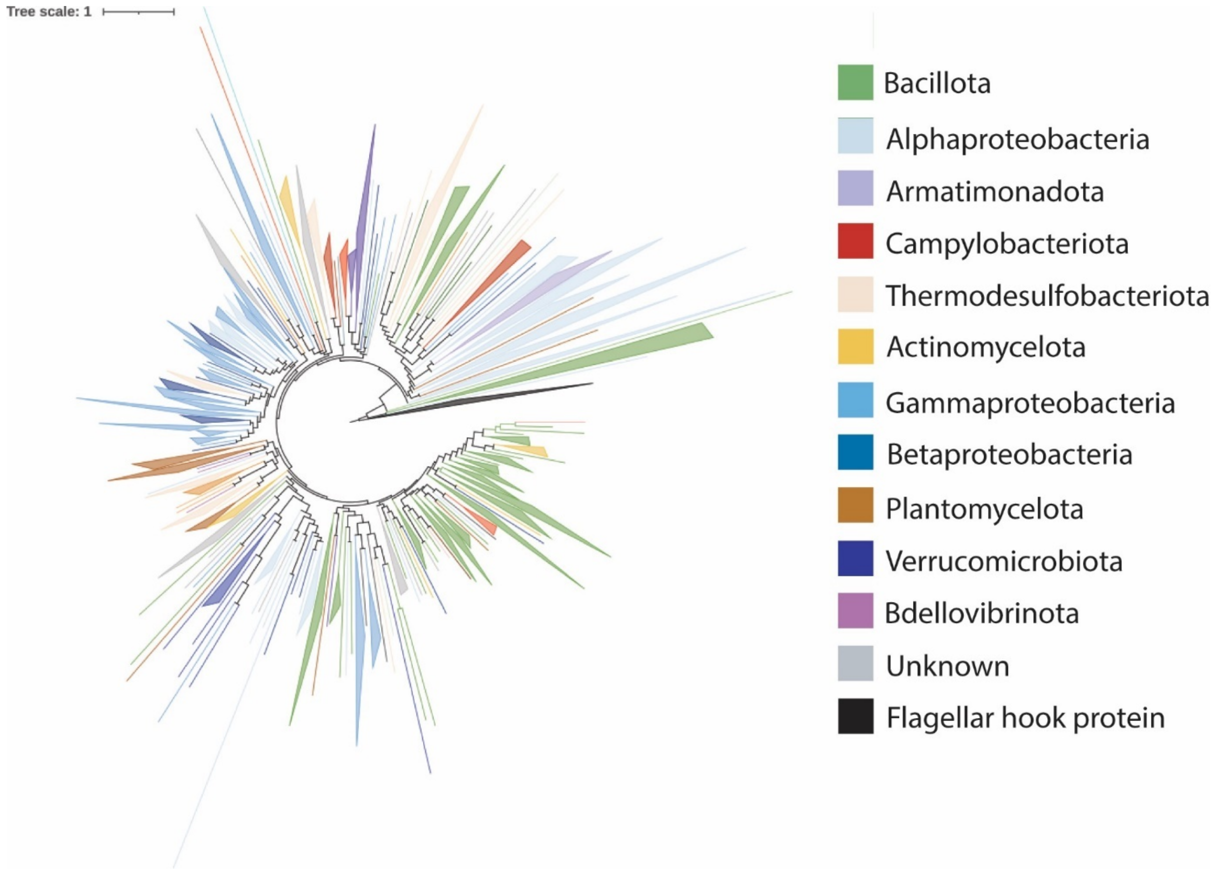

Figure S1. Phylogenetic tree generated using FastTree of 605 available representative flagellin sequences from Fields *et al*., (20). 14 hook-associated protein sequences as the root. Clades were determined if two or more branches from the same phylum/family were adjacent.

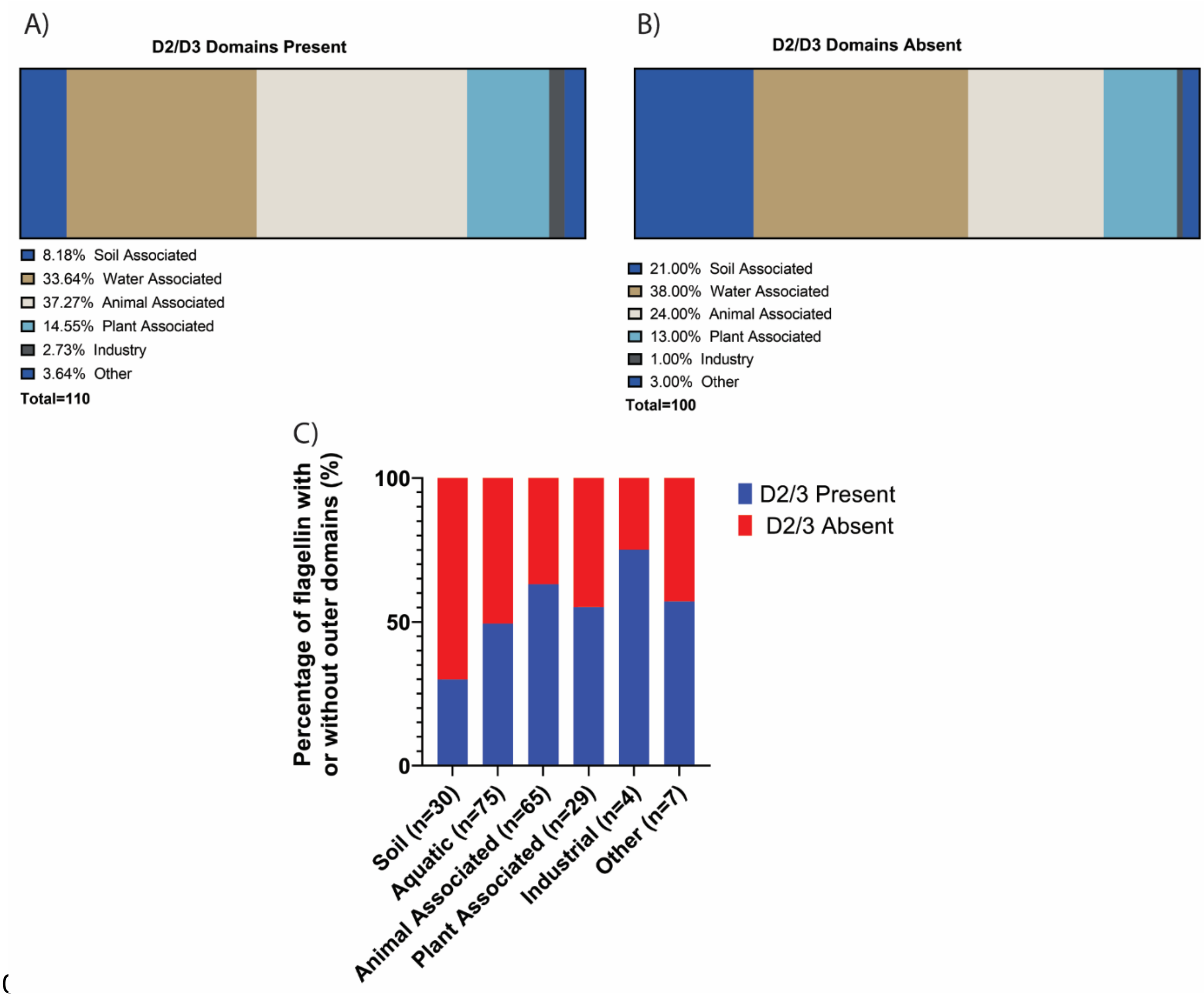

Figure S2. Isolation sources of bacterial flagellins with outer domains A) present and B) absent used to make the flagellin phylogeny. Isolation sources are group in six major clusters; Soil (Blue), Aquatic (Dark Brown), Animal Associated (Light Brown), Plant Associated (Light Blue), Industrial (Grey) and Other (Dark Blue). C) Percentages of flagellins with and without outer domains found from each isolation source.

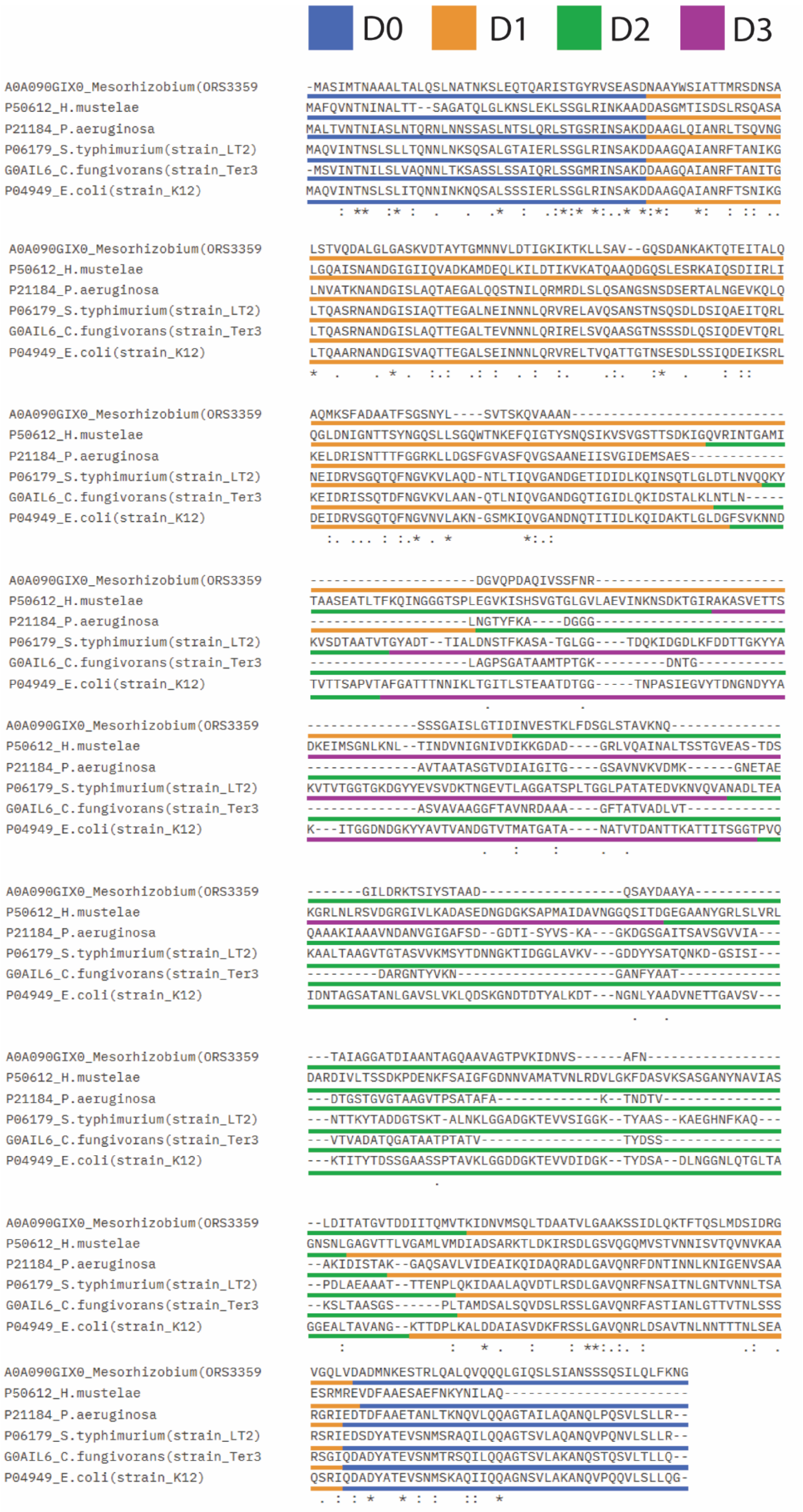
D0 D1 D2 D3
A0A090GIX0_Mesorhizobium(ORS3359 -MASIMTNAAALTALQSLNATNKSLEQTQARISTGYRVSEASDNAAYWSIATTMRSDNSA
P50612_H.mustelae MAFQVNTNINALTT--SAGATQLGLKNSLEKLSSGLRINKAADDASGMTISDSLRSQASA
P21184_P.aeruginosa MALTVNTNIASLNTQRNLNNSSASLNTSLQRLSTGSRINSAKDDAAGLQIANRLTSQVNG
P06179_S.typhimurium(strain_LT2) MAQVINTNSLSLLTQNNLNKSQSALGTAIERLSSGLRINSAKDDAAGQAIANRFTANIKG
G0AIL6_C.fungivorans(strain_Ter3 -MSVINTNILSLVAQNNLTKSASSLSSAIQRLSSGMRINSAKDDAAGQAIANRFTANITG
P04949_E.coli(strain_K12) MAQVINTNSLSLITQNNINKNQSALSSSIERLSSGLRINSAKDDAAGQAIANRFTSNIKG
A0A090GIX0_Mesorhizobium(ORS3359 LSTVQDALGLGASKVDTAYTGMNNVLDTIGKIKTKLLSAV--GQSDANKAKTQTEITALQ
P50612_H.mustelae LGQAISNANDGIGIIQVADKAMDEQLKILDTIKVKATQAAQDGQSLESRKAIQSDIIRLI
P21184_P.aeruginosa LNVATKNANDGISLAQTAEGALQQSTNILQRMRDLSLQSANGSNSDSERTALNGEVKQLQ
P06179_S.typhimurium(strain_LT2) LTQASRNANDGISIAQTTEGALNEINNNLQRVRELAVQSANSTNSQSDLDSIQAEITQRL
G0AIL6_C.fungivorans(strain_Ter3 LTQASRNANDGISLAQTTEGALTEVNNNLQRIRELSVQAASGTNSSSDLQSIQDEVTQRL
P04949_E.coli(strain_K12) LTQAARNANDGISVAQTTEGALSEINNNLQRVRELTVQATTGTNSESDLSSIQDEIKSRL
A0A090GIX0_Mesorhizobium(ORS3359 AQMKSFADAATFSGSNYL----SVTSKQVAAAN--------------------------
P50612_H.mustelae QGLDNIGNTTSYNGQSLLSGQWTNKEFQIGTYSNQSIKVSVGSTTSDKIGQVRINTGAMI
P21184_P.aeruginosa KELDRISNTTTFGGRKLLDGSFGVASFQVGSAANEIISVGIDEMSAES-----------
P06179_S.typhimurium(strain_LT2) NEIDRVSGQTQFNGVKVLAQD-NTLTIQVGANDGETIDIDLKQINSQTLGLDTLNVQQKY
G0AIL6_C.fungivorans(strain_Ter3 KEIDRISSQTDFNGVKVLAAN-QTLNIQVGANDGQTIGIDLQKIDSTALKLNTLN-----
P04949_E.coli(strain_K12) DEIDRVSGQTQFNGVNVLAKN-GSMKIQVGANDNQTITIDLKQIDAKTLGLDGFSVKNND
A0A090GIX0_Mesorhizobium(ORS3359 --------------------DGVQPDAQIVSSFNR------------------------
P50612_H.mustelae TAASEATLTFKQINGGGTSPLEGVKISHSVGTGLGVLAEVINKNSDKTGIRAKASVETTS
P21184_P.aeruginosa -------------------LNGTYFKA----DGGG------------------------
P06179_S.typhimurium(strain_LT2) KVSDTAATVTGYADT--TIALDNSTFKASA-TGLGG----TDQKIDGDLKFDDTTGKYYA
G0AIL6_C.fungivorans(strain_Ter3 -------------------LAGPSGATAAMTPTGK---------DNTG-----------
P04949_E.coli(strain_K12) TVTTSAPVTAFGATTTNNIKLTGITLSTEAATDTGG-----TNPASIEGVYTDNGNDYYA
A0A090GIX0_Mesorhizobium(ORS3359 -------------SSSGAISLGTIDINVESTKLFDSGLSTAVKNQ-------------
P50612_H.mustelae DKEIMSGNLKNL--TINDVNIGNIVDIKKGDAD----GRLVQAINALTSSTGVEAS-TDS
P21184_P.aeruginosa -------------AVTAATASGTVDIAIGITG----GSAVNVKVDMK------GNETAE
P06179_S.typhimurium(strain_LT2) KVTVTGGTGKDGYYEVSVDKTNGEVTLAGGATSPLTGGLPATATEDVKNVQVANADLTEA
G0AIL6_C.fungivorans(strain_Ter3 -------------ASVAVAAGGFTAVNRDAAA----GFTATVADLVT-----------
P04949_E.coli(strain_K12) K---ITGGDNDGKYYAVTVANDGTVTMATGATA----NATVTDANTTKATTITSGGTPVQ
A0A090GIX0_Mesorhizobium(ORS3359 -------GILDRKTSIYSTAAD-----------------QSAYDAAYA-----------
P50612_H.mustelae KGRLNLRSVDGRGIVLKADASEDNGDGKSAPMAIDAVNGGQSITDGEGAANYGRLSLVRL
P21184_P.aeruginosa QAAKIAAAVNDANVGIGAFSD--GDTI-SYVS-KA---GKDGSGAITSAVSGVVIA---
P06179_S.typhimurium(strain_LT2) KAALTAAGVTGTASVVKMSYTDNNGKTIDGGLAVKV---GDDYYSATQNKD-GSISI---
G0AIL6_C.fungivorans(strain_Ter3 ---------DARGNTYVKN-------------------GANFYAAT------------
P04949_E.coli(strain_K12) IDNTAGSATANLGAVSLVKLQDSKGNDTDTYALKDT---NGNLYAADVNETTGAVSV---
A0A090GIX0_Mesorhizobium(ORS3359 ---TAIAGGATDIAANTAGQAAVAGTPVKIDNVS------AFN----------------
P50612_H.mustelae DARDIVLTSSDKPDENKFSAIGFGDNNVAMATVNLRDVLGKFDASVKSASGANYNAVIAS
P21184_P.aeruginosa ---DTGSTGVGTAAGVTPSATAFA------------K--TNDTV---------------
P06179_S.typhimurium(strain_LT2) ---NTTKYTADDGTSKT-ALNKLGGADGKTEVVSIGGK--TYAAS--KAEGHNFKAQ---
G0AIL6_C.fungivorans(strain_Ter3 ---VTVADATQGATAATPTATV-----------------TYDSS--------------
P04949_E.coli(strain_K12) ---KTITYTDSSGAASSPTAVKLGGDDGKTEVVDIDGK--TYDSA--DLNGGNLQTGLTA
A0A090GIX0_Mesorhizobium(ORS3359 --LDITATGVTDDIITQMVTKIDNVMSQLTDAATVLGAAKSSIDLQKTFTQSLMDSIDRG
P50612_H.mustelae GNSNLGAGVTTLVGAMLVMDIADSARKTLDKIRSDLGSVQGQMVSTVNNISVTQVNVKAA
P21184_P.aeruginosa --AKIDISTAK--GAQSAVLVIDEAIKQIDAQRADLGAVQNRFDNTINNLKNIGENVSAA
P06179_S.typhimurium(strain_LT2) --PDLAEAAAT--TTENPLQKIDAALAQVDTLRSDLGAVQNRFNSAITNLGNTVNNLTSA
G0AIL6_C.fungivorans(strain_Ter3 --KSLTAASGS------PLTAMDSALSQVDSLRSSLGAVQNRFASTIANLGTTVTNLSSS
P04949_E.coli(strain_K12) GGEALTAVANG--KTTDPLKALDDAIASVDKFRSSLGAVQNRLDSAVTNLNNTTTNLSEA
A0A090GIX0_Mesorhizobium(ORS3359 VGQLVDADMNKESTRLQALQVQQQLGIQSLSIANSSSQSILQLFKNG
P50612_H.mustelae ESRMREVDFAAESAEFNKYNILAQ-----------------------
P21184_P.aeruginosa RGRIEDTDFAAETANLTKNQVLQQAGTAILAQANQLPQSVLSLLR--
P06179_S.typhimurium(strain_LT2) RSRIEDSDYATEVSNMSRAQILQQAGTSVLAQANQVPQNVLSLLR--
G0AIL6_C.fungivorans(strain_Ter3 RSGIQDADYATEVSNMTRSQILQQAGTSVLAKANQSTQSVLTLLQ--
P04949_E.coli(strain_K12) QSRIQDADYATEVSNMSKAQIIQQAGNSVLAKANQVPQQVLSLLQG-

Figure S3. Alignment of flagellins used to make two and three species FliC chimeras. Colours indicate flagellin domains (Blue = D0, Orange = D1, Green = D2, Purple = D3).

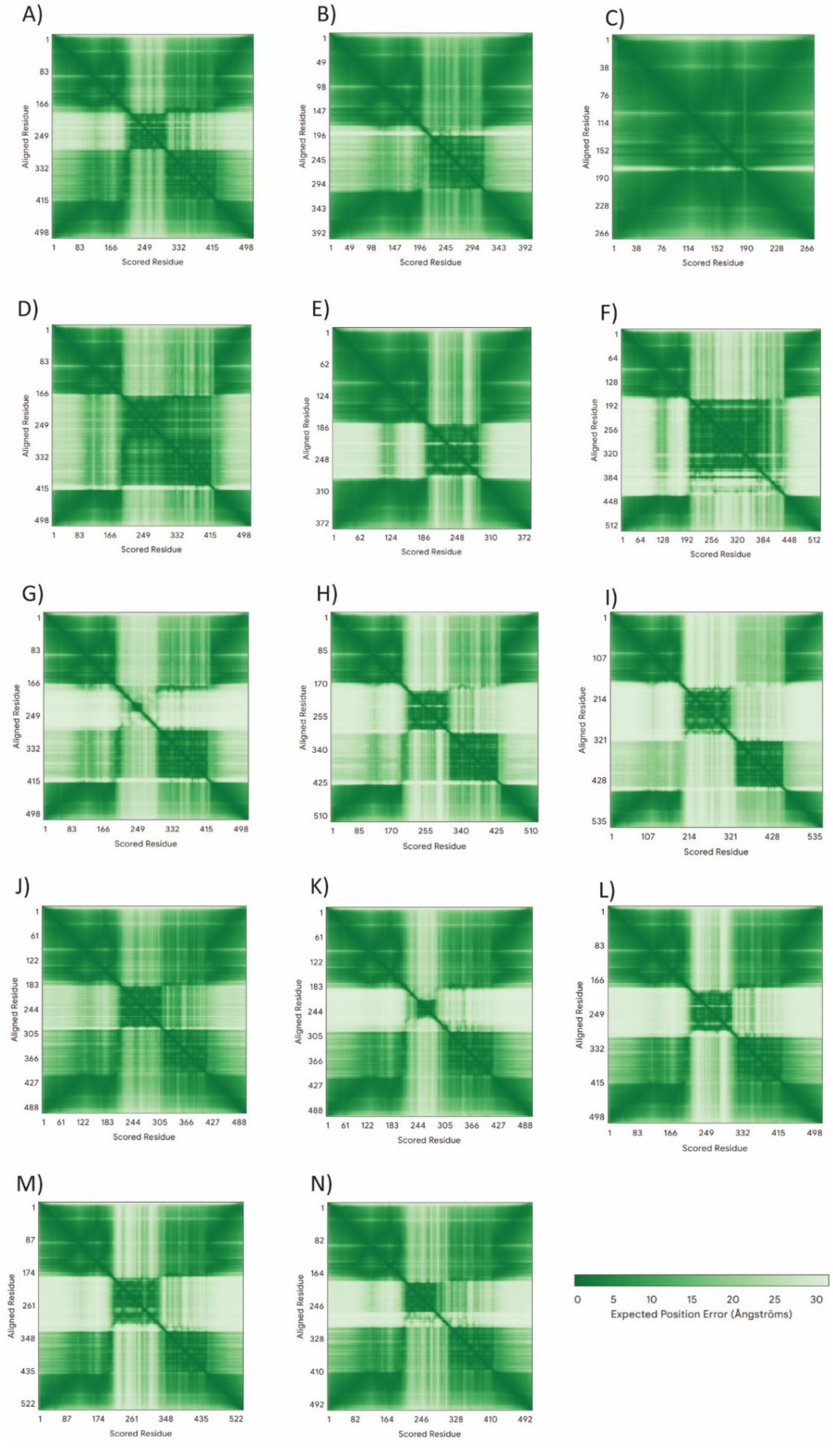

Figure S4. Alphafold3 Predicted Aligned Error heatmaps for *E. coli* K-12 FliC and each FliC mutant. Plots of all AlphaFold3 structural predictions for EEEE, EEE, EE, EESS, EEC, EEHH, EESM, EESC,EESP, EEES, EEEM, EEEC, EEEP and EEEH are represented in panels A-N, respectively.

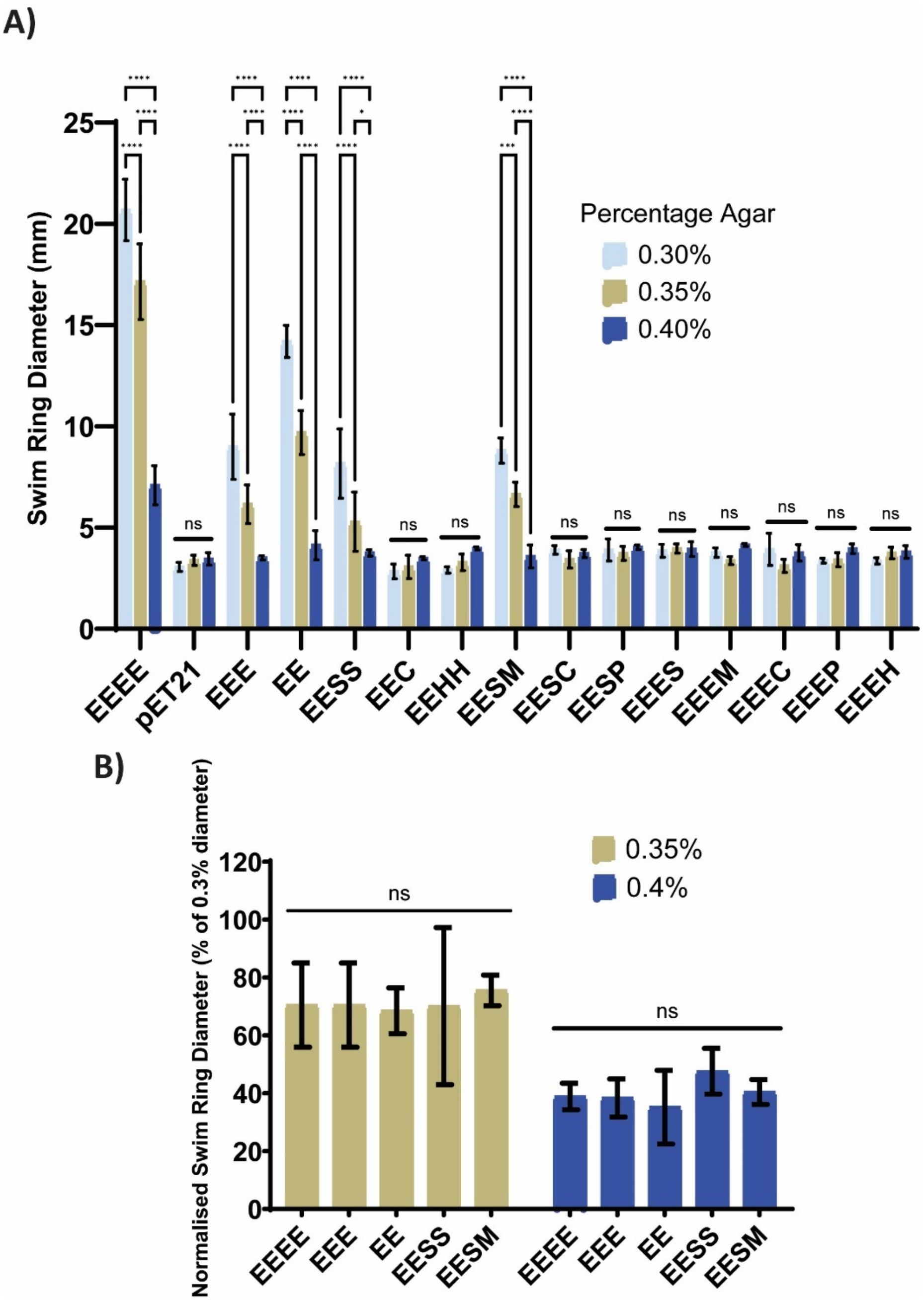

Figure S5. A) Swim ring diameters of chimeric fliC expressing *E. coli* strains in 0.3, 0.35, 0.4 and 0.45% agar swim plates. A two-way ANOVA comparing each column to FliC was used to generate the p values (**** = ≤0.0001, *** = ≤0.001and * = ≤ 0.05). B) Normalised percentage swim ring diameters of motile flagellin variants compared to swim rings from 0.3% agar plates. A two-way ANOVA comparing each column to FliC was used to generate the p values (ns = not significant)

Supplemental Phylogeny S1: Phylogeny in NEXUS format for phylogeny displayed in Figure 1.

Supplemental Phylogeny S2: Phylogeny in NEXUS format for phylogeny displayed in Figure S1.
